# BAP1 tumor predisposition syndrome diagnosed from multiple onychopapillomas

**DOI:** 10.1016/j.jdcr.2026.06.021

**Published:** 2026-06-17

**Authors:** Frank Lin, Yoshine Saito, Russell Newkirk, Isaac Brownell

**Affiliations:** a Department of Dermatology, Walter Reed National Military Medical Center, Bethesda, Maryland; b Dermatology Branch, National Institute of Arthritis and Musculoskeletal and Skin Diseases, NIH, Bethesda, Maryland

**Keywords:** BAP1, BAP1 tumor predisposition syndrome, onychopapilloma

## Introduction

*BRCA1*-associated protein-1 (BAP1) is a ubiquitin carboxy-terminal hydrolase encoded at the chromosome 3q21.3 locus and is known for its tumor suppressor functions.^1^ Germline mutations in *BAP1* result in BAP1 tumor predisposition syndrome (BAP1-TPDS), an autosomal dominant hereditary cancer syndrome. BAP1-TPDS is associated with an increased risk of various benign and malignant tumors, including BAP1-inactivated melanocytoma (BIM) of the skin, cutaneous and uveal melanomas, pleural or peritoneal malignant mesothelioma, and clear-cell renal carcinomas.^2^

Onychopapilloma is a rare, benign tumor of the nail matrix that typically presents as longitudinal leukonychia, erythronychia, or melanonychia and may also exhibit features such as onycholysis, subungual keratosis, and splinter hemorrhage.^3^^,^^4^ It most often presents as a solitary lesion affecting a single digit. However, recently, polydactylous onychopapillomas were described in patients with BAP1-TPDS.^3^^,^^5^

Herein, we report a case of a patient presenting with multiple onychopapillomas, prompting genetic testing which diagnosed a germline *BAP1* mutation.

## Case report

A 43-year-old Caucasian man was found to have multiple white longitudinal streaks on multiple fingernails during a routine full-body skin examination as part of post-melanoma follow-up. He noted the nail changes occurred gradually over years and denied any associated pain, tenderness, or preceding fingernail trauma. Two years prior to presentation, the patient was diagnosed with a Breslow depth 2.4 mm, pT3a N0 M0 (Stage IIA) melanoma of the left forearm which was treated with wide local excision and sentinel lymph node biopsy. His dermatological history was also significant for diffuse actinic keratoses on the forearms, treated with photodynamic therapy, and a persistent melanonychia affecting the right first digit toenail.

Upon directed questioning, the patient’s family history was found to be significant for skin and non-skin cancers: his mother had basal cell carcinoma; his maternal grandfather had malignant mesothelioma, and his 2 maternal great-uncles and a maternal great-aunt had cutaneous melanoma. On the paternal side, his paternal uncle had lung cancer, and his paternal grandfather had liver cancer.

**Fig 1:**
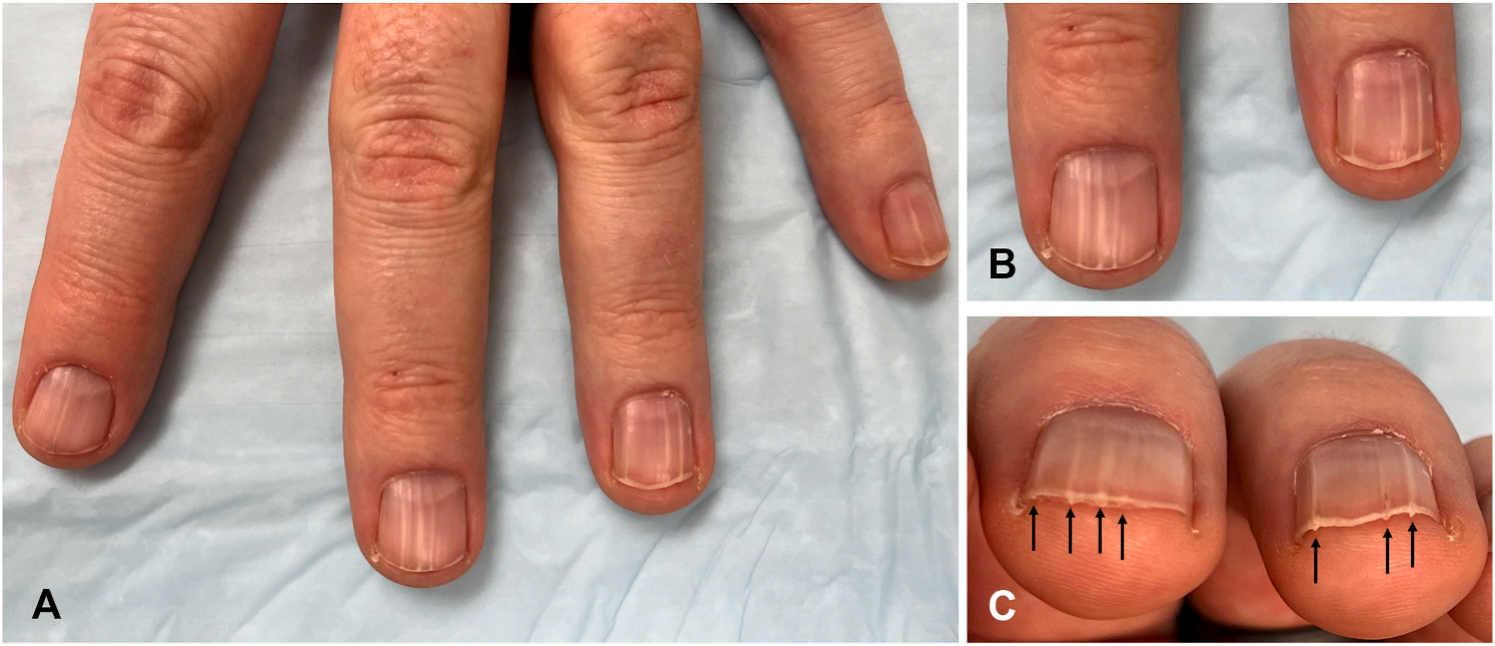
Clinical presentation of onychopapillomas. **A,** Longitudinal leukonychia observed on the left-hand fingernails. Close-up views of the left-hand fingernails digit 2 and 3 showing **(B)** longitudinal leukonychia and **(C)** distal nail subungual hyperkeratosis indicated by *black arrows*.

On physical examination, there were multiple longitudinal leukonychia extending from the proximal nail matrix to the distal edge of the nail on all 10 fingers. Further examination of the hyponychium revealed at least 21 distinct foci of distal nail hyperkeratosis (Fig 1).

Given the polydactylous onychopapillomas, the patient’s personal history of early-onset cutaneous melanoma, and a family history of cutaneous melanoma and mesothelioma, BAP1-TPDS was clinically suspected. The patient was referred to genetic counseling for further evaluation, and genetic testing reveled a pathogenic variant in the *BAP1* gene (GRCh38 chr3:52403428, NM_004656.4:c.1717del), confirming a molecular diagnosis of BAP1-TPDS. This variant has been reported in multiple unrelated individuals with BAP1-TPDS and is classified as pathogenic by at least 9 independent submitters.^6^ However, this variant is not present in the population database gnomAD.

Following his diagnosis of BAP1-TPDS, the patient elected to participate in ongoing *BAP1* related research protocols at the National Institute of Health (NIH) (Protocol numbers 19-C-0049 and 20-C-0106). As part of the study design, he underwent baseline evaluations, including video-assisted thoracoscopic surgery (VATS) as well as diagnostic laparoscopy. Although he had no detectable mesothelial disease on imaging, surgical pathology of biopsies revealed very low-volume *BAP1*-associated mesothelioma in the right pleura, left pleura, and peritoneum. A multidisciplinary tumor board recommended annual imaging surveillance with consideration for repeat VATS procedure in 2 to 3 years, or enrollment in a phase I/II clinical trial investigating oral decitabine/cedazurine for subclinical *BAP1*-associated mesothelioma at the NIH (Protocol number 001549-C). The patient elected to enroll in the drug trial as part of his ongoing disease management.

## Discussion

Currently, there are no formally established clinical diagnostic criteria for BAP1-TPDS. Suggestive indicators of a germline *BAP1* pathogenic variant include either: (1) 2 or more confirmed BAP1-TPDS-associated tumors in a single individual, or (2) 1 BAP1-TPDS tumor and a first- or second-degree relative with a confirmed BAP1-TPDS tumor.^2^^,^^7^ However, reliance on such criteria may lead to delayed diagnosis of BAP1-TPDS, especially considering that some tumors like BIMs and early-stage pleural mesotheliomas can clinically resemble benign processes.^8^ This underscores the need for more specific and sensitive screening strategies for individuals with BAP1-TDPS to enable earlier detection, earlier lifestyle and cancer screening interventions, and potentially better prognostic outcomes.

In addition, given the variable expressivity and incomplete penetrance of BAP1-TPDS, clinical recognition of less common but highly specific findings—such as onychopapillomas—may help identify at-risk individuals who do not yet demonstrate the conventional diagnostic indicators.

As an example of this, onychopapillomas in our patient prompted a targeted family history evaluation that was positive for mesothelioma, supporting subsequent genetic testing. This, in turn, facilitated the early detection of mesothelioma, allowing for timely enrollment in a treatment trial.

Importantly, although the longitudinal leukonychia in our patient was highly suggestive of multiple onychopapillomas, no nail histology was performed. The differential diagnosis for these nail lesions includes onychocytic matricomas, which have also been described in association with BAP1-TPDS.^9^

Once a diagnosis of BAP1-TPDS is established through molecular genetic testing, patients with BAP1-TPDS are typically placed under higher levels of cancer surveillance and counseled on avoiding known environmental risk factors. In the United States, current recommended evaluations and management include: annual full-body dermatology examinations beginning at age 18, annual dilated eye examinations with fundus imaging beginning at age 11-16, and routine clinical examinations combined with abdominal ultrasound or MRI starting at age 30.^7^^,^^8^^,^^10^ Patients are also advised to avoid unnecessary and prolonged sun exposure, routine chest X-ray and CT scans, tobacco use, arc welding, and asbestos exposure.^2^

In summary, this case illustrates how nail abnormalities—specifically polydactylous onychopapillomas—can serve as an early and noninvasive clinical clue pointing toward a germline *BAP1* mutation. Further studies are necessary to evaluate the diagnostic value of onychopapilloma in the context of BAP1-TPDS, particularly in patients with fewer or isolated onychopapillomas. It is also unclear what risk, if any, exists for malignant onychopapilloma or subungual squamous cell carcinoma in BAP1-TPDS.^3^ Nonetheless, increased awareness of the cutaneous and nail manifestations of BAP1-TPDS among primary care providers and dermatologists may facilitate earlier diagnosis and intervention in this cancer predisposition syndrome.

## Conflicts of interest

None disclosed.
